# Aging, Work, and Multicultural Populations: Findings From National Data

**DOI:** 10.1093/geroni/igaf122.1077

**Published:** 2025-12-31

**Authors:** Rebecca Perron

**Affiliations:** AARP, Washington, District of Columbia, United States

## Abstract

AARP is committed to the ongoing study of older workers, including within multicultural populations. A 2025 nationally representative study of older workers in the US authored by AARP revealed persistently high rates of age discrimination for African American/Black (74%), Hispanic/Latino (62%), and Asian American and Pacific Islander (67%) workers ages 50-plus. A large majority of these groups report that age discrimination is common. Beyond the more common or overt forms of age discrimination – like not getting a job due to age - subtle forms are also prevalent. These include assumptions that older workers are less tech-savvy, comments on their health related to age, beliefs that they are resistant to change, and inquiries about when they might retire. Furthermore this study found that around 2 in 5 older workers from multicultural backgrounds feel they are being pushed out of their job due to their age. Additional nationally representative data from AARP found that significant proportions of multicultural older workers expect to change jobs in the next year. Specifically, two in five of African American and Hispanic and 33% of AAPI employed older workers expect to make a job change. Money is the primary motivator of job change for all groups. Very large majorities recognize the need for some sort of help or information as they change jobs. Despite concerns about the impact of artificial intelligence on job security and age being a barrier to finding new employment, these groups remain confident in their ability to successfully change jobs.

